# Comprehensive analysis of the endoplasmic reticulum stress-related long non-coding RNA in bladder cancer

**DOI:** 10.3389/fonc.2022.951631

**Published:** 2022-08-04

**Authors:** Zhenyu Wu, Yue Wang, Mengxin Yan, Quan Liang, Bin Li, Guoliang Hou, Taolin Xia, Zhe Lin, Wenfeng Xu

**Affiliations:** ^1^ Department of Urology, The First People’s Hospital of Foshan, Foshan, China; ^2^ The First Clinical Medical College, GuangDong Medical University, ZhanJiang, China

**Keywords:** bladder cancer, TCGA, lncRNA, prognostic model, ERS

## Abstract

**Background:**

Bladder cancer is ranked the second most frequent tumor among urological malignancies. The research strived to establish a prognostic model based on endoplasmic reticulum stress (ERS)-related long non-coding RNA (lncRNA) in bladder cancer.

**Methods:**

We extracted the ERS-related genes from the published research and bladder cancer data from the Cancer Genome Atlas database. ERS-related lncRNAs with prognostic significance were screened by univariate Cox regression, least absolute shrinkage and selection operator regression analysis and Kaplan-Meier method. Multivariate Cox analysis was leveraged to establish the risk score model. Moreover, an independent dataset, GSE31684, was used to validate the model’s efficacy. The nomogram was constructed based on the risk score and clinical variables. Furthermore, the biological functions, gene mutations, and immune landscape were investigated to uncover the underlying mechanisms of the ERS-related signature. Finally, we employed external datasets (GSE55433 and GSE89006) and qRT-PCR to investigate the expression profile of these lncRNAs in bladder cancer tissues and cells.

**Results:**

Six ERS-related lncRNAs were identified to be closely coupled with patients’ prognosis. On this foundation, a risk score model was created to generate the risk score for each patient. The ERS-related risk score was shown to be an independent prognostic factor. And the results of GSE31684 dataset also supported this conclusion. Then, a nomogram was constructed based on risk scores and clinical characteristics, and proven to have excellent predictive value. Moreover, the gene function analysis demonstrated that ERS-related lncRNAs were closely linked to fatty extracellular matrix, cytokines, cell adhesion, and tumor pathways. Further analysis revealed the association of the 6-lncRNAs signature with gene mutations and immunity in bladder cancer. Finally, the external datasets and qRT-PCR verified high expressions of the ERS-related lncRNAs in bladder cancer tissues and cells.

**Conclusions:**

Overall, our findings indicated that ERS-related lncRNAs, which may affect tumor pathogenesis in a number of ways, might be exploited to assess the prognosis of bladder cancer patients.

## Introduction

As the world’s number ten most frequent malignant tumor and number two most prevalent urological malignancy, bladder cancer has caused a total of 213,000 deaths and 573,000 new cases in 2020 (1). Patients with bladder cancer receive a prognosis that is closely linked to the pathological diagnosis, comprising non-muscle invasive bladder cancer (NMIBC) as well as muscle invasive bladder cancer (MIBC). Most patients with NMIBC eventually progress to MIBC, whose 5-year overall survival (OS) was less than 50% (2, 3). Even with proper treatment, approximately half of bladder cancer patients suffer from recurrence or distant metastasis after radical surgery (4, 5). However, prognosis prediction and individualized treatment of bladder cancer still remain a challenge as it may be relevant to the biological heterogeneity of tumor (6). Because current approaches are insufficient for reliably assessing the prognosis of bladder cancer, a more trustworthy method must be investigated.

Endoplasmic reticulum stress (ERS) is defined as an imbalance in endoplasmic reticulum homeostasis, which includes the unfolded protein response (UPR) and perturbation in calcium (7). Relevant studies have demonstrated that ERS has a major part in the genesis and progression of a variety of human malignancies (8). Sustained mobilization of ERS endows malignant cells with greater tumorigenic, metastatic and drug resistant capacities (9). However, ERS overactivation may disrupt cellular homeostasis and leads to tumor cell death  (10). On account for this, ERS and their downstream signaling pathways have emerged as a key regulator of tumor progression and response to chemotherapy and immunotherapy (11).

Recently, emerging studies have indicated that long non-coding RNA (lncRNA) plays a significant role in the regulation of gene expression and contributes to the development of numerous human diseases (12). Abnormalities of lncRNAs have been verified to be tumor-suppressive or tumor-oncogenic and to play a key role in tumor development (13). LncRNAs are important in multi-gene regulatory networks, and they can be utilized to diagnose and predict survival in a variety of cancers (14). Multiple lncRNAs signature have been researched in bladder cancer (15–17). However, the role of ERS-related lncRNA in bladder cancer remains to be studied.

In this research, we extracted the sequencing data and corresponding clinical data from the Cancer Genome Atlas (TCGA) and ERS-related genes from published research. We strived to establish a prognostic model based on ERS-related lncRNA, which can evaluate the prognosis of bladder cancer patients, and to explore its function in the occurrence and development of bladder cancer.

## Materials and methods

### Identification of ERS-related LncRNAs

We downloaded the RNA sequencing data, gene mutation data and corresponding clinical information of bladder cancer from TCGA database^1^, and extracted an ERS-related genes from published literature (18). On the premise that correlation coefficient |R^2^| > 0.3 and *p* < 0.05 were considered to be statistically significant, we identified ERS-related lncRNAs through Pearson correlation analysis. In the condition of |log_2_ (Fold Change)| > 1 and False Discovery Rate < 0.05, we obtained the ERS-related lncRNAs differentially expressed between tumor and normal adjacent tissues (NATs).

### Screening of prognosis-related LncRNAs

Patients with follow-up of more than one month were included in the following study. Univariate Cox regression was performed to identify prognosis-associated lncRNAs with a criteria of *p* < 0.05. Least absolute shrinkage and selection operator (LASSO) regression analysis and Kaplan-Meier analysis were used to further screened lncRNAs tightly related to OS.

### Building a risk score model

Patients were randomly classified into the training and testing sets in a 2:1 ratio. In the training set, we executed multivariate stepwise Cox regression on the above lncRNA to compute their respective coefficients (βi). Then, a risk score model was constructed from βi and gene expression levels (Expi). According to the formula, the risk score for each patient is calculated. In addition, we used the median risk score as a cutoff to classify all eligible patients into high- and low-risk teams. Prognostic differences between high- and low-risk patients were revealed using Kaplan-Meier survival curves, and we applied the testing group to validate the above findings. Meanwhile, we collected the GSE31684 dataset (n = 93) from the Gene Expression Omnibus (GEO) database^2^ to verify the model’s efficacy.

### Constructing and evaluating nomogram

In the training set, we used univariate and multivariate Cox regression analyses to estimate the prognostic significance of risk scores and clinical factors, including gender, age, and TMN stage. In order to predict OS of each patient, risk scores and clinical characteristics were incorporated to establish a nomogram. Next, the area under the curve (AUC) values were acquired by receiver operating characteristic (ROC) curves to evaluate the nomogram’s predictive efficacy. Subsequently, the predictability of this nomogram was verified by concordance index (C-index) method, calibration plots, and testing cohort.

### Functional analysis

We performed gene set enrichment analysis (GSEA) on the gene expression profile between the high- and low-risk cohorts using the gene Ontology (GO) and Kyoto Encyclopedia of Genes and Genomics (KEGG) gene sets. The molecular mechanism and biological functions of risk score may provide insights into the underlying mechanisms of ERS-related lncRNAs. Besides, we analyzed the gene mutation status between two groups to explore possible links between ERS-related lncRNAs and mutations. To explore the relationship between these lncRNAs and immunity, the immune cell abundance, immune function, and immune checkpoints were compared between two cohorts. Furthermore, the immunophenoscore was used to predict response to immunotherapy between two groups, which were acquired from the Cancer Immunome Atlas^3^ (19).

### External datasets and qRT-PCR validation

GSE89006 dataset (4 pairs of bladder cancer and NATs) and GSE55433 dataset (tumor = 57, normal = 26) were collected in the GEO database to verify the expression of target lncRNAs.

Next, bladder cancer cells (EJ-1, U3, and 5637) and normal bladder epithelium cells (SV-HUC-1) were purchased from Cell Bank, Institute of Life Sciences, Chinese Academy of Sciences Cell Bank (Shanghai, China). Total RNA was isolated using TRIzol reagent (Bioteke, Beijing, China). Then, reverse transcription was conducted utilizing the HiScript II Q RT SuperMix reagent (Vazyme, Nanjing, China). Subsequently, qRT-PCR was performed by the Hieff TMqPCR SYBR^®^ Green Master Mix (Yeasen, Shanghai, China) with the primers provided in the **Supplementary Table S1**. We executed qRT-PCR with the Bio-Rad CFX96 Real-time Quantitative PCR System (Bio-Rad, California, USA).

### Statistical analysis

Statistical analyses of this research were entirely conducted in the R software^4^. To identify the relationships between ERS-related genes and lncRNAs, Pearson correlation analysis was carried out. For comparison of the differences of categorical and continuous variables, the Chi-square test as well as the t-test was used, respectively. Univariate Cox regression, multivariate Cox regression, LASSO regression analysis, and Kaplan-Meier method were conducted to identify the optimal prognostic factors. In evaluating the OS of patients in various groups, the Kaplan-Meier method was utilized, and differences between groups were assessed utilizing log-rank test. At a two-tailed *p* < 0.05, the results were regarded statistically significant.

## Results

### Identification of ERS-related LncRNAs

**Figure 1:**
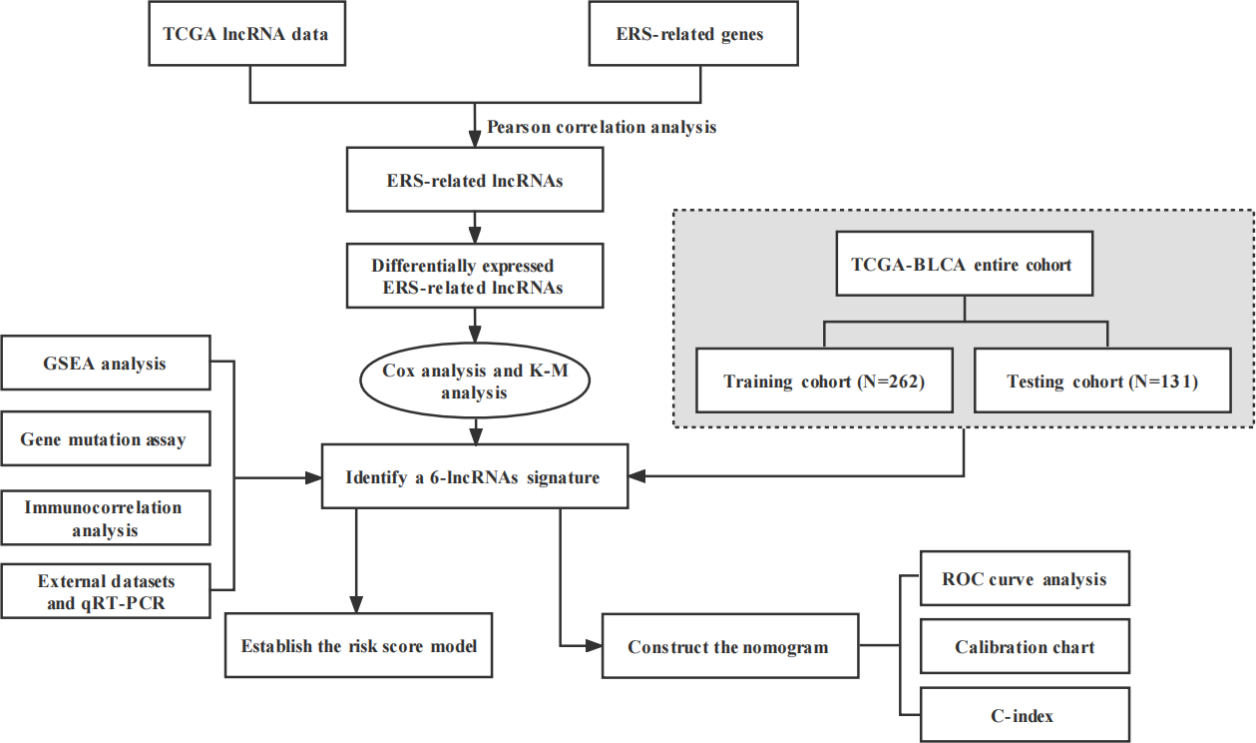
This study’s design and flowchart.

**Figure 2:**
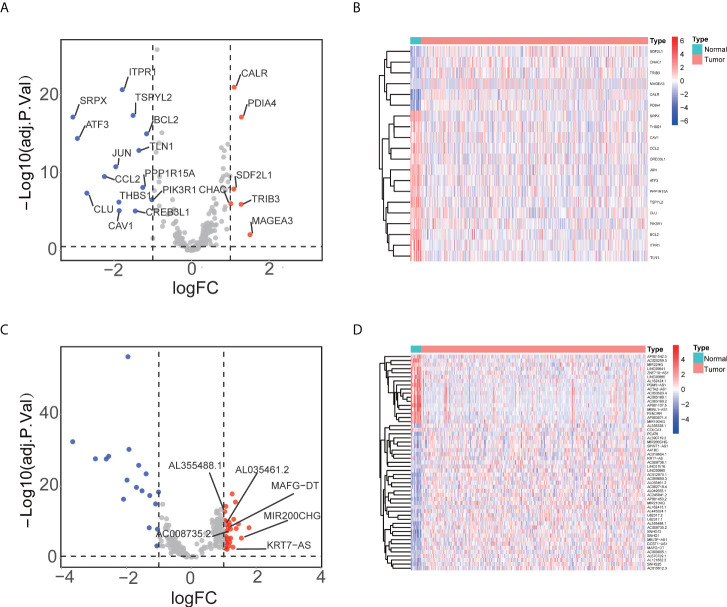
Identifying ERS-related lncRNA in tumor. **(A)** The volcano plot revealed that in tumors, 6 ERS-related genes were up-regulated whereas 14 were down-regulated. **(B)** The heatmap showed expression profile of ERS-related genes. **(C)** The volcano plot visualized that in tumors, 35 ERS-related lncRNAs were up-regulated and 18 were down-regulated. **(D)** The heatmap illustrated expression profile of ERS-related lncRNAs.

**Figure 1** presented the flowchart of our investigation. We obtained data of 411 tumor tissues and 19 NATs from 411 patients in TCGA database, and 252 ERS-related genes from the published research (**Supplementary Table S2**). A total of 263 ERS-related lncRNAs were identified with Pearson correlation analysis. Then, we screened out 53 ERS-related lncRNAs that were differentially expressed in bladder cancer and NATs, including 18 upregulated and 35 downregulated in tumors (**Figure 2**).

### Screening of prognosis-associated LncRNAs

**Figure 3:**
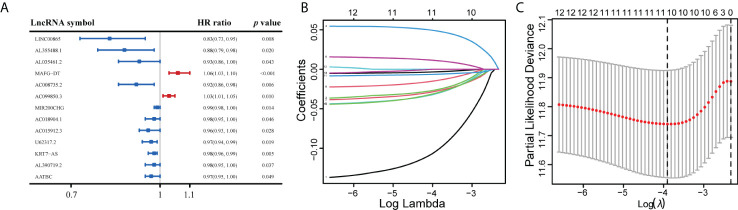
Identifying ERS-related lncRNAs associated to OS. **(A)** The forest plot recognized 13 ERS-related lncRNAs substantially associated with OS. **(B)** Lasso regression analysis. **(C)** The LASSO coefficient spectrum for lncRNA associated with OS was presented.

We performed univariate Cox regression on the above ERS-related lncRNAs and recognized 13 lncRNAs associated with prognosis (**Figure 3A**). Six key lncRNAs were further screened by LASSO analysis (**Figure 3B, C**) and Kaplan-Meier analysis (**Supplementary Figure S1**), namely AL355488.1, AL035461.2, MAFG-DT, AC008735.2, MIR200CHG, and KRT7-AS.

### Establishing a risk scoring model

**Figure 4:**
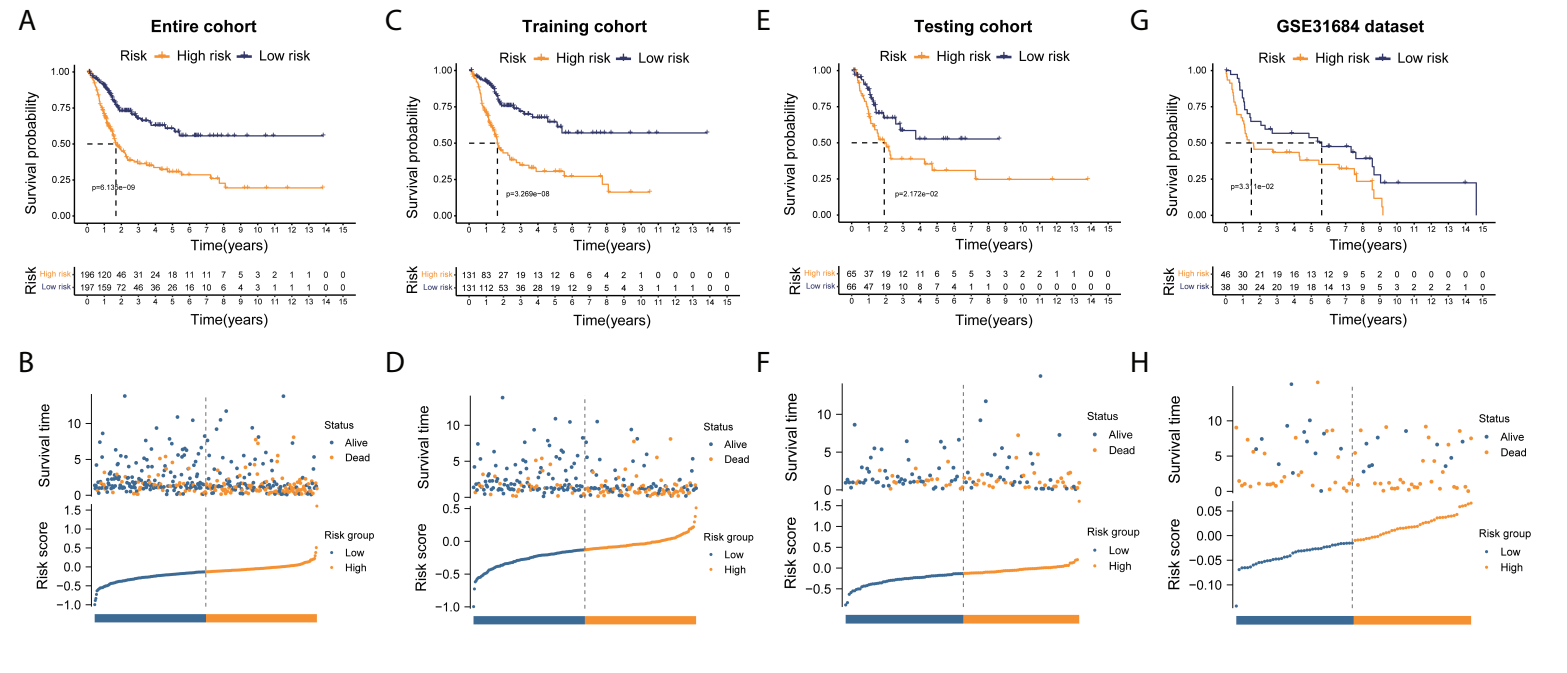
Risk score model establishment utilizing ERS-related lncRNAs. **(A, C, E, G)** The Kaplan-Meier analysis unveiled a significantly shorter OS of the high-risk group than that of the low-risk group in entire, training, testing cohorts, and GSE31684 dataset, respectively. **(B, D, F, H)** The overview of each patient’s survival status and risk rating distributions in entire, training, testing cohorts, and GSE31684 dataset, respectively.

In the training set, we constructed a risk score model including Expi and βi: $\text{Risk score}=\sum_{i=1}^{5}\left(Exp_{i}*\beta_{i}\right)$ (**Table 1**). Patients were split into two groups, a high-risk group and a low-risk group, according to the median risk score. The high-risk group’s OS was considerably shorter in comparison to the low-risk group (**Figure 4**). Comparing to the risk coefficient and the mortality of patients of the low-risk group, those of the high-risk group were higher (**Figure 4D**). Similar findings were revealed using the same method on the testing group (**Figure 4E, F**). Meanwhile, the results of GSE31684 dataset also verified the model’s efficacy (**Figure 4G, H**).

**Table 1:** The prognostic significance of the 6-lncRNAs signature.

ERS-related lncRNAs	Coef	HR
AL355488.1	-0.024623135	0.881333378
AL035461.2	-0.018783391	0.926879906
MAFG-DT	0.044346228	1.062089764
AC008735.2	-0.017581442	0.917739074
MIR200CHG	-0.002668355	0.987752228
KRT7-AS	-0.003720336	0.976742661

### Constructing and evaluating nomogram

**Figure 5:**
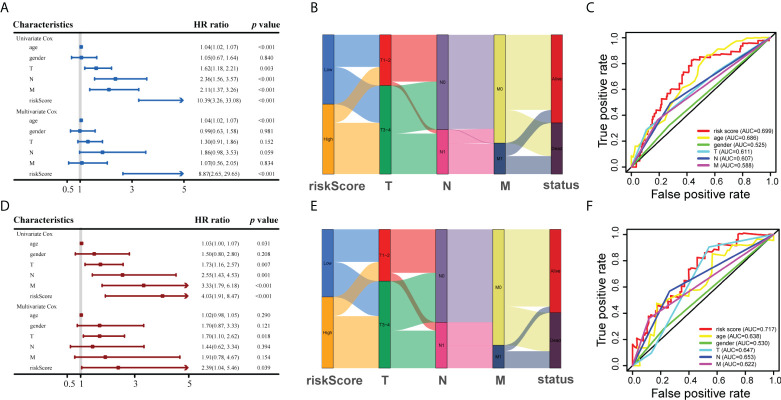
Analyses of prognostic values using the risk score and clinical variables. **(A, D)** Risk score was revealed to be strongly associated to OS in the univariate Cox analysis and multivariate Cox analysis in training and testing cohort, respectively. **(B, E)** Alluvial diagrams showing the associations between risk scores and clinical variables in training and testing groups, respectively. **(C, F)** ROC curve analyses of the risk scores and clinical variables in training and testing sets, respectively.

**Figure 6:**
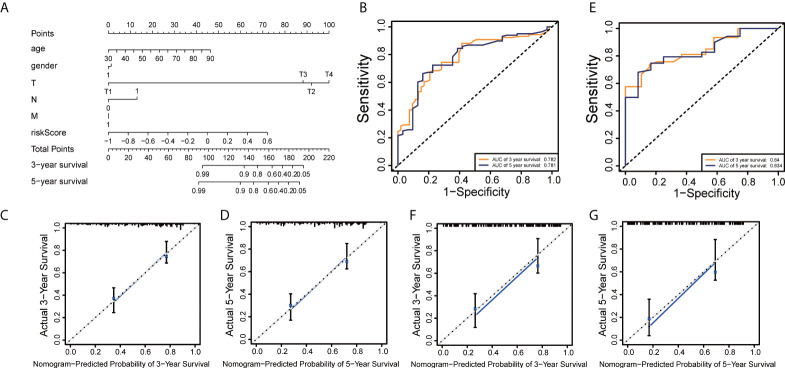
Establishment and interpretation of the nomogram for clinical variables and ERS-related lncRNAs. **(A)** Establishment of the nomogram. **(B, E)** ROC curve analyses of the nomogram in training and testing sets, respectively. **(C, F)** The nomogram’s 3-year OS calibration curve in training and testing groups, respectively. **(D, G)** The nomogram’s calibration curve for a 5-year OS in training and testing sets, respectively.

In the training set, it was observed that the clinical variables and risk score were closely associated to OS in the univariate Cox regression. Further multivariate Cox analysis indicated that risk score was an independent prognostic factor (**Figure 5A, B**). In addition, the ROC curve illustrated that the 6-lncRNAs signature was a remarkable prognostic predictor (**Figure 5C**). Then, using the multivariate Cox regression results, including clinical factors and risk scores, we constructed a nomogram (**Figure 6A**). Then we analyzed the 3- and 5-year OS of this prognostic model using ROC curves and obtained AUCs of 0.782 and 0.781, respectively (**Figure 6B**). The C-index of the model was 0.742. The established calibration plots demonstrated that the model had a favorable predictive effect (**Figure 6C, D**). We obtained similar outcomes with the same technique on the testing cohort (**Figure 5D–F**, **6E–G**). The C-index of testing cohort was 0.717.

### Functional analysis

**Figure 7:**
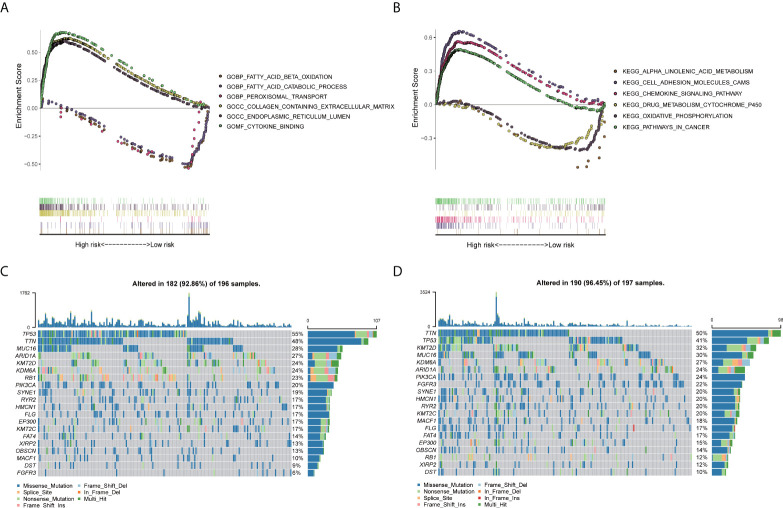
The results of GSEA and gene mutation analyses. **(A)** According to GO enrichment analysis, ERS-related lncRNAs were may be involved in fatty acid metabolism, peroxidation, extracellular matrix, endoplasmic reticulum, and cytokines. **(B)** KEGG pathway analysis showed that these lncRNAs may be associated with functions such as cell adhesion, cytokines, drug metabolism, tumor pathways, and oxidative phosphorylation. **(C, D)** Waterfall plots of gene somatic mutations in high- and low-risk patients, respectively.

**Figure 8:**
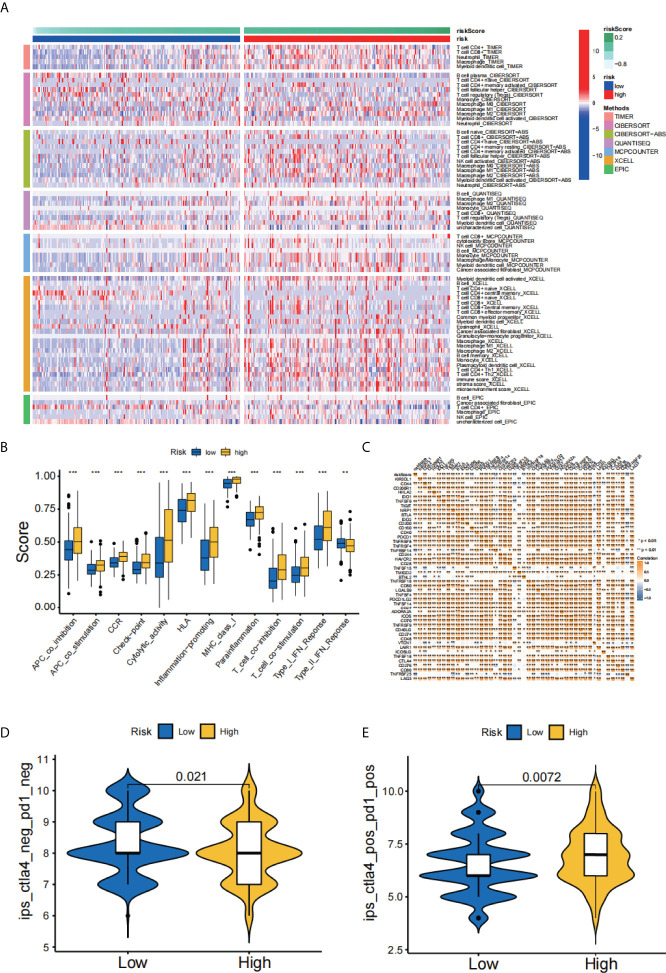
Analysis of immune landscape. **(A)** A heatmap for immunological responses between high- and low-risk groups. **(B)** Immune cell subpopulations and associated functions. **(C)** Expression of immune checkpoints in high- and low-risk cohorts. **(D, E)** Relative response of anti-PD-1 and anti-CTLA4 therapy between two groups. **p < 0.01; ***p < 0.001.

ERS-related lncRNAs may be involved in fatty acid metabolism, peroxidation, extracellular matrix, endoplasmic reticulum, and cytokines (**Figure 7A**), which were demonstrated by GO analysis. KEGG analysis indicated that these lncRNAs may be associated with functions such as cell adhesion, cytokines, drug metabolism, tumor pathways, and oxidative phosphorylation (**Figure 7B**). Gene mutations between the high-risk patients and the low-risk patients were displayed in waterfall plots (**Figures 7C, D**). The top five genes with the highest mutation frequency in the high-risk team were TP53, TTN, MUC16, ARID1A, and KMT2D, while the top five genes in the low-risk team were TTN, TP53, KMT2D, MUC16, and KDM6A, revealing ERS-related lncRNAs may be associated with gene mutations. As it was shown in **Figure 8A**, the abundances of CD4+ T cells, CD8+ T cells, neutrophils, and macrophages were markedly enriched in the high-risk team compared to the low-risk team. Immune functions including cytolytic activity, HLA function, IFN response, and T cell stimulation were relatively active in the high-risk group (**Figure 8B**). In addition, the study of immune checkpoints uncovered a high expression of immunosuppressive receptors (CTLA4, PD-1, LAG3, BTLA, and TIGIT) and immunosuppressive ligands (PD-L2 and TNFSF14) in high-risk cohort (**Figure 8C**). Further analysis showed that the response to immunotherapy in the high-risk set was better than that in the low-risk set, suggesting that risk scores can predict the efficacy of immunotherapy (**Figure 8D, E**). Overall, the association between the risk score and tumor immune landscape were assessed, and the outcomes demonstrated that the risk score was related to different immune landscape.

### External datasets and qRT-PCR validation

**Figure 9:**
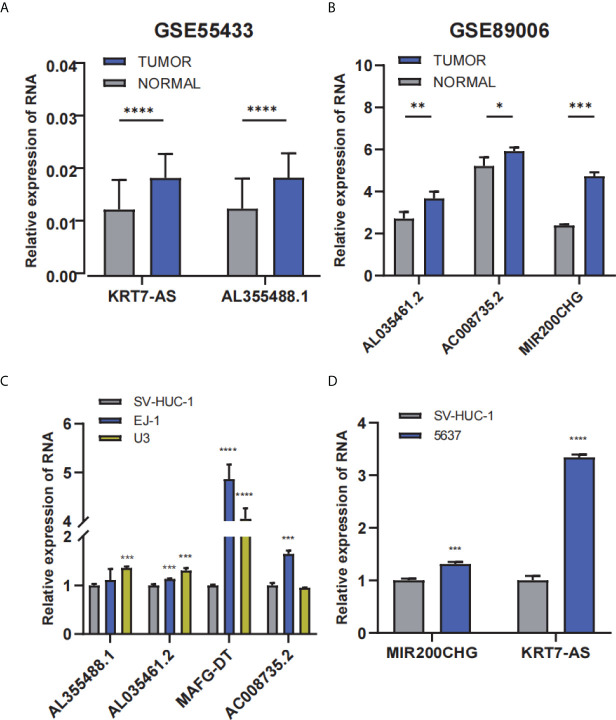
The expression profile of the 6-lncRNAs signature with external validation. **(A, B)** The lncRNA expression profile in GSE55433 and GSE89006 datasets, respectively. **(C, D)** The lncRNA expression level in bladder cancer cells and normal cells. *p < 0.05; **p < 0.01; ***p < 0.001; ****p < 0.0001.

Next, we validated the expression level of target lncRNAs using the GSE55433 and GSE89006 datasets. The GSE55433 dataset discovered that KRT7-AS and AL355488.1 were significantly overexpressed in tumor compared with NATs (**Figure 9A**). Furthermore, GSE89006 dataset showed a high expression of AL035461, AC008735.2, and MIR200CHG in bladder cancer compared with NATs (**Figure 9B**).

Moreover, qRT-PCR showed that the expression of AL355488.1, AL035461.2, MAFG-DT, and AC008735.2 was significantly upregulated in EJ-1 and U3 cells compared with SV-HUC-1 cells (**Figure 9C**). In 5637 cell line, MIR200CHG and KRT7-AS were considerably upregulated (**Figure 9D**). The results of external datasets and qRT-PCR were consistent with the findings of TCGA analysis.

## Discussion

Bladder cancer is one of the most common malignancies of the genitourinary system, with approximately 550,000 new cases each year (20). The high incidence, malignant behavior, and drug resistance of bladder cancer have made the therapy and prognostic management an increasing challenge (21). The search for a novel molecular marker to assess the prognosis and guide the treatment of patients with bladder cancer is therefore of great importance.

Accumulated studies have developed multiple signatures to predict prognosis of patients with tumors. Huang et al. (18) uncovered a ERS-related signature which may serve as a marker for prognosis prediction and individualized therapy of glioma. Zhou et al. (22) revealed that the immune-related lncRNAs signature had prognostic value for glioblastoma patients. Zheng et al. (23) have developed a hypoxia-immune signature to predict the prognosis and immune status for triple-negative breast cancer. Xie et al. (24) constructed a necroptosis signature to predict prognosis and drug sensitivity of triple-negative breast cancer. Meanwhile, a ferroptosis-based signature has been established, which may serve as a novel therapy biomarker for lung squamous cell carcinoma (25). However, the role of ERS-related lncRNA in bladder cancer remains to be studied.

In this study, we extracted ERS-related genes and sequencing data of bladder cancer from online database, and obtained ERS-related lncRNAs differentially expressed between tumor and NATs. Next, we further screened prognosis-associated lncRNAs by univariate Cox regression, LASSO regression and Kaplan-Meier analyses. The 6-lncRNAs signature was utilized to develop a risk model that separated patients of bladder cancer into a high-risk group and a low-risk group. Prognosis analysis revealed that the low-risk group patients presented favorable OS. Additionally, a nomogram was established and demonstrated to have a strong prognostic effect.

Through our study, 6 lncRNAs were identified to be associated with bladder cancer prognosis. Among them, AL355488.1, AL035461.2, MIR200CHG, AC008735.2, and KRT7-AS had a protective effect for prognosis, while MAFG-DT presented the opposite effect. AL355488.1 has previously been shown to be a potential biomarker for targeted therapy and prognosis in hepatocellular liver cancer (26). And MIR200CHG was found to be a protective indicator for bladder cancer patients (27). AC008735.2 was significantly associated with targeted therapy and prognosis in patients with clear cell renal cell carcinoma (28). KRT7-AS played an important role in cancer regulation and was superior to other clinicopathological features in predicting survival (29). In addition, the expression of MAFG-DT in bladder cancer was higher than that in NATs and can promote tumor growth and progression, which can be served as a predictor of patient prognosis (30).

In GSEA analysis, these ERS-related lncRNAs were associated with functions of fatty acid metabolism, peroxidation, extracellular matrix, cytokines, cell adhesion, tumor pathways, and oxidative phosphorylation. It has been shown that fatty acid metabolism may be associated with the production and stemness-maintenance of cancer stem cell, thereby affecting the prognosis of tumors (31, 32). Besides, extracellular matrix regulation could not only facilitate tumor cells invasion but also contribute to the generation and maintenance of cancer stem cell niche (33). Moreover, the regulation of cell adhesion alters the ability of tumor cells to interact with extracellular matrix and adjacent cells, thereby affecting the biological behavior of tumors (34). It was generally known that the endoplasmic reticulum was the origin of ERS, and the degradation of stress proteins helped restore cellular homeostasis (35, 36). Taken together, these findings may inspire scholars to further explore the molecular mechanism of ERS-related lncRNAs in bladder cancer.

In addition, we analyzed the mutations between the high- and low-risk patients. The top five mutated genes in the high-risk cohort were TP53, TTN, MUC16, ARID1A, and KMT2D, while those in the low-risk cohort were TTN, TP53, KMT2D, MUC16, and KDM6A. It was reported that TP53 mutation can increase cancer cells’ resistance to ERS through maintaining the activation of UPR regulator ATF6 and inhibition of pro-apoptotic factors JNK and CHOP (37). This led us to speculate that increased TP53 mutations in high-risk team may inhibit the ERS damage in tumor cells, resulting in a poorer prognosis of patients. Similarly, ARID1A mutation was demonstrated to protect cancer cells from changes in tumor microenvironment by upregulating ERS (38). It made us hypothesize that elevated ARID1A mutations in the high-risk team might promote tumor progression and bring about poor prognosis. Furthermore, the investigation of immune landscape showed that the infiltration of immune cell and immune functions in the high-risk team were relatively active compared to those in the low-risk team. Notably, the infiltration of CD8+ T cells was significantly higher in the high-risk group than in the low-risk group, which contradicted the antitumor effect of these cells. On this basis, we further explored the immune checkpoints and uncovered a high expression of immunosuppressive receptors (CTLA4, PD-1, LAG3, BTLA, and TIGIT) and immunosuppressive ligands (PD-L2 and TNFSF14) in high-risk cohort. Overexpression of these molecules might promote immune tolerance in bladder cancer, thereby affecting the prognosis of patients. The elucidation of immune checkpoints could also help identify potential target patients and contribute to the development of immunotherapy.

Our research has a few limitations. For starters, it is just a retrospective research based on lncRNA data and few clinical variables from TCGA database, which is lacking in some detailed clinical information. Second, the prognostic value and the biological functions of ERS-related lncRNAs are not yet fully elucidated. Finally, although the validation was performed in the cell lines through qRT-PCR, further *in vivo* or *in vitro* experiments were needed to investigate the concrete mechanisms of the lncRNAs.

## Conclusion

We constructed a prognostic model in the basis of ERS-related lncRNA, which had a superior effect on predicting the survival rate of patients with bladder cancer. Therefore, this indicated that ERS-related lncRNAs can be a reliable predictor of the prognosis for bladder cancer patients and may be closely related to the occurrence and development of bladder cancer.

## Data availability statement

The original contributions presented in the study are included in the article/**Supplementary Material**. Further inquiries can be directed to the corresponding authors.

## Ethics statement

All data of our research were derived from TCGA and GEO database and required no ethical approval.

## Author contributions

WX and ZL designed and facilitated the study. ZW and YW analyzed the data and completed the draft. MY and QL participated in the revision of the manuscript. BL, GH, and TX collected the data. All authors read the manuscript and concur with its submission.

## Funding

This work was funded by the Foshan Ascending Peak Plan Project (grant no. 2020A012), Guangdong Basic and Applied Basic Research Foundation (grant no. 2021A1515111197), and Guangdong Medical Scientific Research Foundation (grant no. A2022222).

## Acknowledgments

We thank the researchers and patients who contributed data to TCGA and GEO database.

## Conflict of interest

The authors declare that the research was conducted in the absence of any commercial or financial relationships that could be construed as a potential conflict of interest.

## Publisher’s note

All claims expressed in this article are solely those of the authors and do not necessarily represent those of their affiliated organizations, or those of the publisher, the editors and the reviewers. Any product that may be evaluated in this article, or claim that may be made by its manufacturer, is not guaranteed or endorsed by the publisher.

## Footnotes

1
https://cancergenome.nih.gov/

2
https://www.ncbi.nlm.nih.gov/gds/

3
https://tcia.at/

4
https://www.r-project.org, version 4.0.2
