# Supplementary material for: Comprehensive analysis of the endoplasmic reticulum stress-related long non-coding RNA in bladder cancer
Source: Front Oncol. 2022 Aug 4;12:951631. doi: 10.3389/fonc.2022.951631 (PMC9386564; doi:10.3389/fonc.2022.951631)
Supplement: Supplementary file 1 [file DataSheet_1.docx]

Supplementary Material

**Supplementary Table S1. The primer sequences of ERS-related lncRNAs**

| Symbol | Sequence (5’-3’) |
| --- | --- |
| B-catin-QPCR-H-F | TGGCACCCAGCACAATGAA |
| B-catin-QPCR-H-R | CTAAGTCATAGTCCGCCTAGAAGCA |
| AL355488.1-QPCR-F | AGCAGCAGTTGGGTTTGGAT |
| AL355488.1-QPCR-R | GTCCCCACACCTCCTCTGTA |
| AL035461.2-QPCR-F | ATATGCCTGTGGTTCCAGCTAC |
| AL035461.2-QPCR-R | ACATTGCACTCCACATAGACTCG |
| MAFG-DT-QPCR-F | AGTGTCTCAAGCCTATAATCCCAG |
| MAFG-DT-QPCR-R | ACAAGCAATCCTCCCACGTC |
| AC008735.2-QPCR-F | ATGAAGAGACAGAGCACACA |
| AC008735.2-QPCR-R | TGCTACATTTGATAAACCAGCC |
| MIR200CHG-QPCR-F | CGGTGACAGTAACCTTCAGGG |
| MIR200CHG-QPCR-R | TTCCCATTGTTCCCCTTTGCC |
| KRT7-AS-QPCR-F | CTTGGCACGAGCATCCTTGA |
| KRT7-AS-QPCR-R | TTAAGCAAGTTGCCCGGGTG |

**Supplementary Table S2. Summary of ERS-related genes**

| ERS-related gene | | | |
| --- | --- | --- | --- |
| ACADVL | DNAJB14 | MBTPS1 | SSR1 |
| ADD1 | DNAJB2 | MBTPS2 | STC2 |
| AGR2 | DNAJB9 | NCK1 | STT3B |
| AIFM1 | DNAJC10 | NCK2 | STUB1 |
| ALOX15 | DNAJC18 | NFE2L1 | SULT1A3 |
| AMFR | DNAJC3 | NFE2L2 | SVIP |
| ANKS4B | EDEM1 | NPLOC4 | SYVN1 |
| ANKZF1 | EDEM2 | NRBF2 | TARDBP |
| APAF1 | EDEM3 | OPA1 | TATDN2 |
| ARFGAP1 | EEF2 | OS9 | TBL2 |
| ASNS | EIF2AK2 | P4HB | THBS1 |
| ATF3 | EIF2AK3 | PARK7 | THBS4 |
| ATF4 | EIF2AK4 | PARP16 | TLN1 |
| ATF6 | EIF2B5 | PDIA2 | TMBIM6 |
| ATF6B | EIF2S1 | PDIA3 | TMCO1 |
| ATG10 | EP300 | PDIA4 | TMEM117 |
| ATP2A1 | ERLEC1 | PDIA5 | TMEM129 |
| ATP2A2 | ERLIN1 | PDIA6 | TMEM33 |
| ATP6V0D1 | ERLIN2 | PDX1 | TMEM67 |
| ATXN3 | ERN1 | PIK3R1 | TMTC3 |
| AUP1 | ERN2 | PIK3R2 | TMUB1 |
| BAK1 | ERP27 | PLA2G6 | TMUB2 |
| BAX | ERP29 | PMAIP1 | TMX1 |
| BBC3 | ERP44 | PML | TNFRSF10B |
| BCAP31 | EXTL1 | POMT2 | TOR1A |
| BCL2 | EXTL2 | PPP1R15A | TP53 |
| BCL2L1 | EXTL3 | PPP1R15B | TPP1 |
| BCL2L11 | FAF2 | PPP2CB | TRAF2 |
| BFAR | FBXO2 | PPP2R5B | TRIB3 |
| BHLHA15 | FBXO6 | PREB | TRIM13 |
| BOK | FCGR2B | PSMC6 | TRIM25 |
| BRSK2 | FGF21 | PTPN1 | TSPYL2 |
| CALR | FICD | PTPN2 | TTC23L |
| CASP4 | FKBP14 | RASGRF1 | TXNDC12 |
| CAV1 | FLOT1 | RASGRF2 | UBA5 |
| CCDC47 | FOXRED2 | RCN3 | UBAC2 |
| CCL2 | GET4 | RHBDD1 | UBE2G2 |
| CCND1 | GFPT1 | RNF103 | UBE2J1 |
| CDK5RAP3 | GORASP2 | RNF121 | UBE2J2 |
| CEBPB | GOSR2 | RNF139 | UBE2K |
| CFTR | GRINA | RNF175 | UBE4A |
| CHAC1 | GSK3A | RNF183 | UBE4B |
| CLU | GSK3B | RNF185 | UBQLN1 |
| COPS5 | HDGF | RNF186 | UBQLN2 |
| CREB3 | HERPUD1 | RNF5 | UBXN1 |
| CREB3L1 | HM13 | RNFT1 | UBXN4 |
| CREB3L2 | HSP90B1 | SCAMP5 | UBXN6 |
| CREB3L3 | HSPA13 | SDF2 | UBXN8 |
| CREB3L4 | HSPA1A | SDF2L1 | UFC1 |
| CTDSP2 | HSPA5 | SEC16A | UFM1 |
| CTH | HYOU1 | SEC31A | UGGT1 |
| CUL7 | IGFBP1 | SEC61B | UGGT2 |
| CXXC1 | ITPR1 | SEL1L | USP13 |
| DAB2IP | JKAMP | SERINC3 | USP14 |
| DCTN1 | JUN | SERP1 | USP19 |
| DDIT3 | KDELR3 | SERP2 | USP25 |
| DDRGK1 | KLHDC3 | SESN2 | VAPB |
| DDX11 | LMNA | SGTA | VCP |
| DERL1 | LRRK2 | SGTB | WFS1 |
| DERL2 | MAGEA3 | SHC1 | WIPI1 |
| DERL3 | MAN1B1 | SIRT1 | YIF1A |
| DNAJB11 | MANF | SRPRB | YOD1 |
| DNAJB12 | MAP3K5 | SRPX | ZBTB17 |

## Supplementary Figure S1


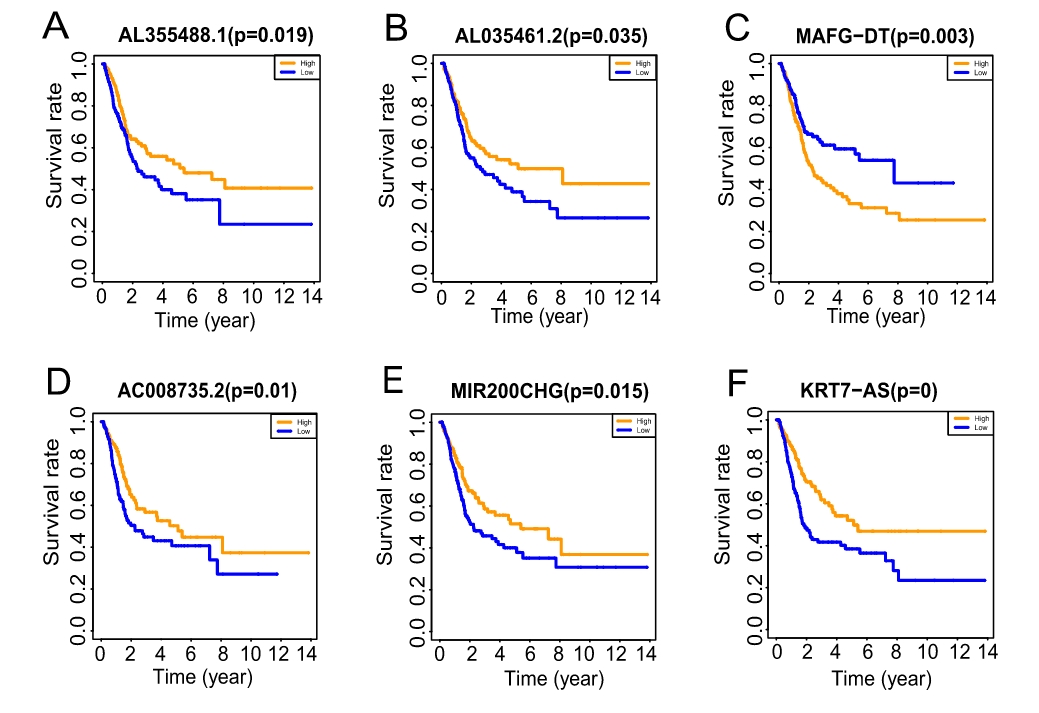


**Supplementary Figure S1.** Survival analyses of lncRNAs associated with OS: AL355488.1 (A), AL035461.2 (B), MAFG-DT (C), AC008735.2 (D), MIR200CHG (E), and KRT7-AS (F).
